# S01-2: Developing a systems-based national physical activity strategy for Scotland

**DOI:** 10.1093/eurpub/ckae114.198

**Published:** 2024-09-26

**Authors:** Jennifer Love

**Affiliations:** Scottish Government

## Abstract

**Purpose:**

To share learning from the application of a pragmatic, evidence-driven, systems-based approach to physical activity policy in Scotland from a Scottish Government perspective.

**Development:**

Scottish Government recognise the need for a systems-based approach to address physical inactivity. GAPPA and the ISPAH 8 investments provide a strong basis for global action but it was essential that we translate these recommendations into our national context. Our public health agency, Public Health Scotland (PHS) undertook this task and provided a framework on which to build a national strategy.

**Implementation and dissemination:**

The development of our new strategy was overseen by a National Leadership Group, chaired by our Minister for Sport included senior leaders from national delivery agencies in transport, education, health, environment and sport, with representation from local authorities and Scotland’s Chief Medical Officer. The purpose of the group is to foster partnership working between agencies and to achieve commitment at both national and local level to improving levels of physical activity in Scotland.

**Evaluation:**

We evaluate the impact of our policies through indicators linked to our delivery outcomes. Data are largely derived from two annual national surveys: The Scottish Health Survey and the Scottish Household Survey

**Conclusions and practical implications:**

Adoption of a systems-based approach derived from international evidence and best practice gives us confidence that our new strategy represents the best approach to improving levels of physical activity in Scotland.

The systems-based approach highlights the importance of achieving commitment across Ministerial portfolios to achieving our outcomes. This can be challenging and having a lead Minister who is personally enthusiastic and focussed on the importance of addressing physical inactivity has been crucial.

Both nationally and locally, Scotland invests significantly in physical activity. The importance of the systems-based approach is that it provides us with a conceptual framework which allows us to better co-ordinate and target existing funding across portfolios to focus on evidence-based action which will deliver our outcomes.

